# Cuckoo Algorithm Based on Global Feedback

**DOI:** 10.1155/2023/2040866

**Published:** 2023-01-07

**Authors:** Xingyu Liu, Tao Wu, Wuxing Lai, Hu Yuan, Qilong Kou, Jingping Yu

**Affiliations:** ^1^School of Software, Huazhong University of Science and Technology, Wuhan 430074, China; ^2^School of Mechanical Science and Engineering, Huazhong University of Science and Technology, Wuhan 430074, China; ^3^Tencent Technology (Shenzhen) Co. Ltd., Shenzhen 518000, China

## Abstract

This article proposes a cuckoo algorithm (GFCS) based on the global feedback strategy and innovatively introduces a “re-fly” mechanism. In GFCS, the process of the algorithm is adjusted and controlled by a dynamic global variable, and the dynamic global parameter also serves as an indicator of whether the algorithm has fallen into a local optimum. According to the change of the global optimum value of the algorithm in each round, the dynamic global variable value is adjusted to optimize the algorithm. In addition, we set new formulas for the other main parameters, which are also adjusted by the dynamic global variable as the algorithm progresses. When the algorithm converges prematurely and falls into a local optimum, the current optimum is retained, and the algorithm is initialized and re-executed to find a better value. We define the previous process as “re-fly.” To verify the effectiveness of GFCS, we conducted extensive experiments on the CEC2013 test suite. The experimental results show that the GFCS algorithm has better performance compared to other algorithms when considering the quality of the obtained solution.

## 1. Introduction

Swarm intelligence algorithm is an algorithm designed to simulate the behavior of natural biological groups, which have been extensively applied for solving complex and highly nonlinear optimization problems. As an emerging optimization algorithm, swarm intelligence algorithm has become one of the focuses of increasingly researchers. Researchers have proposed a variety of algorithms, such as ant colony algorithm (ACO) [1], differential evolution (DE) [2], particle swarm optimization algorithm [3] (PSO), artificial bee colony algorithm [4] (ABC), firefly algorithm [5] (FA), and cuckoo search algorithm [6] (CS). At present, these algorithms have been applied to a variety of engineering optimization problems and have a potential research value. Hence, it is still a promising domain to develop more effective swarm intelligence algorithms.

The CS algorithm, inspired by the parasitic brooding behavior of cuckoos, was proposed by Yang and Deb et al. in 2009. This parasitic behavior has become a breeding strategy for cuckoos, and in most cases, they lay their eggs in the nests of other bird species. Therefore, the host bird may discover that the egg is not its own, at which point it either throws away the foreign egg or abandons the nest and builds a new one. In addition, the CS algorithm employs methods such as greedy selection, random walk, and Lévy flight [7] to solve the global optimal solution. Compared with the uniform distribution and Gaussian distribution algorithm, the long-hop mode algorithm provided by the Lévy flight can search the solution domain better. The combination of Lévy flight advantage and local search ability makes the CS algorithm one of the most effective optimization algorithms. Compared with other swarm intelligence algorithms, CS has the advantages of fewer parameters, simple operation, and strong optimization ability, and it is more effective in solving optimization problems. However, on the contrary, there are also the defects of unbalanced exploration ability and mining ability, and it is easy to fall into the local optimal solution.

Since similar search strategies, Lévy flight and random walk strategies, are adopted in most CSs, the search behaviors of cuckoos are similar, which can easily lead the algorithm to fall into a local optimum and enter premature convergence. Sometimes, the algorithm converges to a local optimum at a very early stage, but the whole algorithm ends without obtaining a better fitness value. Under these circumstances, it not only is difficult to obtain a better value but also wastes subsequent computing resources.

Based on this situation, a new type of cuckoo algorithm (GFCS) is proposed. GFCS dynamically adjusts the parameters of the algorithm according to whether each round of the algorithm iteration produces a better fitness value. In the case where the fitness value remains unchanged for a long time, the current optimal value is retained; then, the algorithm will be reset, resulting in better algorithm performance for the same computational generation. Briefly, the core idea of this work is as follows:

The GFCS algorithm is a CS algorithm which employs random walk and Lévy flight to search for the global optimum. We have proposed three innovations based on the original CS algorithm:

- We introduce the concept of global feedback to adjust the dynamic global variables by the current round of iterations and determine whether the algorithm falls into a local optimum.
- The fixed parameter pattern of the original CS algorithm is optimized. We set the parameter formulas that vary with the number of iteration rounds and is controlled by the dynamic global variables.
- We introduced the “re-fly” mechanism. When the algorithm falls into the local optimum, the algorithm can save the current global optimum value and the algorithm will be initialized and re-executed to find a better value.

The article is organized as follows.  Section 2 reviews the original CS and its technical details. In Section 3, the literature on CS and its application to optimization problems are presented.  Section 4 elaborates on the proposed algorithm. A comparative analysis of numerical experiments between GFCS and CS, multiple CS variants, and several other state-of-the-art algorithms is presented in Section 5. Finally, in Section 6, we summarize the proposed algorithm.

## 2. Basic Cuckoo Search Algorithm

The cuckoo search algorithm (CS) is a swarm intelligence algorithm inspired by the natural behavior of some cuckoo species laying their eggs in other birds' nests. Different from other algorithms, the search process of CS is divided into two stages: global search and local search, corresponding to exploration and exploitation, respectively. The global stage is carried out by the Lévy flight, as the Lévy distribution has infinite mean and variance, which helps to explore the solution space efficiently. The local phase is executed by using the biased random walk.

In the CS algorithm, the number of hosts available is constant. In each iteration, each cuckoo lays only one egg and then randomly places the egg into the host's nest. Each egg is used as a solution to the problem. The host bird has a probability of *Pa* (*Pa* ∈ [0,1]) to find the cuckoo laying eggs in its nest. When this happens, the laid eggs are thrown away or the host bird simply abandons the nest to make a new one.

It is assumed that *N* is the number of cuckoos and *D* represents the dimension of problem, the position of *i* th cuckoo is denoted as *Xi*, and *t* represents the current iteration. Then, the new position can be generated by the following equations:

(1) $$\begin{array}{c} x_{i}^{t+1}=x_{i}^{t}+\alpha ⊕L\text{é}vy\left(s,\lambda \right)\text{,} \end{array}$$

where *α* is the step size, which should be related to the scale of the problem, and the product ⊕ represents the multiplication of the corresponding position of the vector. Therefore, the formula for the Lévy flight is as follows:

(2) $$\begin{array}{c} L\text{é}vy\left(s,\lambda \right)=\frac{\lambda \Gamma \left(\lambda \right)\cdot \sin\;\left(\pi \lambda /2\right)}{\pi }\frac{1}{s^{1+\lambda }}\text{,} \end{array}$$

where

(3) $$\begin{array}{c} \;\alpha =\alpha_{0}\left(x_{i}^{t}-x_{best}^{t}\right)\text{,} \end{array}$$

where *x*_best_^*t*^ represents the optimal solution of the *t*th generation, Lévy(*s*, *λ*) represents the feature scale, *λ* represents the power coefficient (1 < *λ* < 3), and Γ represents the gamma function. In addition, *α*_0_ represents the scaling factor, which controls the size of the step.

In formula (1) and formula (2), s represents the step size of the Lévy flight, and it was designed by Mantegna's algorithm [8] as follows:

(4) $$\begin{array}{c} s=\frac{\mu }{{\left|\nu \right|}^{1/\lambda }}\text{,} \end{array}$$

where *μ* and *ν* are random numbers drawn from a normal distribution:

(5) $$\begin{array}{c} \mu ∼N\left(0,{\sigma_{\mu }}^{2}\right),\nu ∼N\left(0,{\sigma_{\nu }}^{2}\right)\text{,} \end{array}$$

where the value of *σ*_*ν*_ is usually set to 1, and the formula for *σ*_*μ*_ is shown as follows:

(6) $$\begin{array}{c} \sigma_{\mu }={\left\{\frac{\Gamma \left(1+\lambda \right)\cdot \sin\;\left(\pi \lambda /2\right)}{\Gamma \left[\left(1+\lambda \right)/2\right]\cdot \lambda \cdot 2^{\left(\lambda -1\right)/2}}\right\}}^{1/\lambda }\text{.} \end{array}$$

Then, the formula for the local random walk can be expressed as

(7) $$\begin{array}{c} x_{i}^{t+1}=\left\{\begin{array}{cc} x_{i}^{t}+r\cdot \left(x_{j}^{t}-x_{k}^{t}\right)\text{,} & rand>P_{a}\text{,}\\ x_{i}^{t}\text{,} & otherwise\text{.} \end{array}\right. \end{array}$$

where r is a scaling factor uniformly distributed in the range [0, 1] and *x*_*j*_^*t*^ and *x*_*k*_^*t*^ represent two different solutions randomly selected in the population.

Based on the previous introduction, the original CS algorithm framework is shown in Algorithm 1.

## 3. Related Works

The main advantages of the CS algorithm are few parameters, simple operation, easy implementation, optimal random search path, and strong search ability. At present, scholars at home and abroad have also proposed many improvement strategies for the cuckoo algorithm. The main research directions in previous years include improving the step size of the Lévy flight and the random walk algorithms, or adjusting the parameter *Pa* by introducing a new *Pa* formula, or setting a new step size adjustment formula to adjust the performance of the algorithm.

Valian et al. proposed an improved cuckoo algorithm [9] for reliability optimization problems. The optimization of the step size of the Lévy flight was introduced into the algorithm, and the probability of cuckoo eggs being found *Pa* was adjusted. With the change of the number of iterations, the step size alpha and *Pa* were gradually reduced according to a certain formula. Naik et al. proposed a new cuckoo algorithm [10] that abandoned the Lévy flight by using a step size based on the number of iterations, the contemporary optimal nest, the contemporary worst nest, and the average nest fitness value. Ong proposed an adaptive cuckoo algorithm [11], which compared the current fitness value with the average fitness value and used different step size algorithms according to the comparison results to ensure that the algorithm had a faster convergence speed in the early stage and a large convergence accuracy in the later stage. Wang et al. proposed a cuckoo algorithm with different scale factors [12]. During the iteration process, random numbers were introduced to make fluctuations at each step, which improved the performance of the algorithm but reduced the stability of the algorithm. Li and Yin proposed a modified cuckoo search algorithm with a self-adaptive parameter method [13]. A linear change of parameters was achieved by introducing the ratio of the current algebra to the total algebra, and according to the success rate of the evolution of the previous generation, different schemes of *Pa* were selected. Huang et al. proposed a cuckoo search (CS) algorithm using an elite opposition-based strategy [14], in which the proposed algorithm generated the opposite solutions of elite individuals in the population by an opposition-based strategy. The algorithm was guided to explore the optimal solution by simultaneously evaluating the current population and the opposite population. Based on the elite opposition-based strategy mentioned previously, Baset et al. proposed a new cuckoo search algorithm [15] for solving integer programming problems, which had faster convergence and higher computational accuracy and was more effective.

Some scholars adjusted or replaced the Lévy flight with some new models, such as introducing other algorithms or introducing Gaussian functions to speed up the optimization. Kamoona et al. proposed an enhanced cuckoo algorithm [16], which replaced the Lévy flight with the Gaussian virus diffusion idea, and innovatively introduced the search formula of the artificial bee colony algorithm. Zheng and Zhou proposed a new cuckoo algorithm based on Gaussian distribution optimization [17]. The algorithm replaced the Lévy flight with the Gaussian distribution to a certain extent, and the algorithm had relatively good performance in local optimization performance. He et al. proposed a Spark-based Gaussian Bare-bones cuckoo Search with dynamic parameter selection [18]. For *Pa* values, a pool of candidate *Pa* values in the range [0.01, 0.5] was introduced, and the value for the step size was generated by a Gaussian distribution.

Inspired by the organizational evolutionary algorithm for numerical optimization, Zheng and Zhou designed a novel algorithm, the cooperative co-evolutionary cuckoo search algorithm (CCCS) [19], which combined dynamic populations and evolutionary operators for solving both unconstrained, constrained optimization and engineering problems. Cheng and Wang proposed a neighborhood-attracting cuckoo algorithm [20], which introduced the concept of neighborhood in the cuckoo algorithm, making the fitness value of the bird's nest closer to the best bird's nest in the area. At the same time, the difference between the fitness value of the individual and the best individual was analysed, and the step size of this iteration was determined by the difference.

Some researchers tried to optimize the cuckoo algorithm by mixing multiple algorithms, randomly or based on certain feedback to select the most suitable algorithm for the current generation, which diversified the iteration selection of the algorithm. The disadvantage was that while promoting the algorithm's search ability, it also increased the instability of the algorithm. Rakhshani and Rahati proposed a new cuckoo algorithm based on Snap-Drift [21]. The algorithm divided the entire optimization process into two modes: snap and drift, selecting the best search mode according to the optimization effect of the current algebra. Peng et al. proposed a multistrategy serial cuckoo algorithm [22], which divided the execution process of the overall algorithm into three stages, including jump learning, Gaussian walking learning, and begging behavior. Different strategies were formulated at each stage and achieved better results. In addition, he also proposed another similar algorithm, the multistrategy reconciliatory cuckoo search algorithm [23], which updated individuals based on a harmonic strategy. The adaptive step size guided the cuckoo to seek optimization in a better direction, and three improved update methods were explored analytically from three perspectives: their own neighborhood, the current optimal individual, and the random position. Gao et al. proposed a multistrategy adaptive cuckoo algorithm [24], and the algorithm was designed with five different search ideas. According to the performance of each iteration, a certain selection ratio was set for these five strategies, making the algorithm tend to be diversified.

In addition, the cuckoo algorithm also has some other improvements. Zhang et al. proposed a dynamic adaptive cuckoo search algorithm [25], which introduced feedback into the algorithm framework and established a closed-loop control system for CS algorithm parameters. The improvement rate was maintained at 20 percent, and *Pa* and *α* were dynamically adjusted. Walton et al. proposed an adjusted cuckoo algorithm [26] to classify bird nests. The excellent parts were set as the top nests; then, new nests were constructed by the relationship between the top nests. Excellent nests were used to construct new nests, thus selecting the best ones and improving the local exploration ability of the algorithm, while the poor nests used a larger step size to improve the global ability of the algorithm.

At the same time, the cuckoo algorithm and its improved algorithm also have a very broad application, such as PID controller design [27], grayscale image enhancement [28], cryptanalysis of Vigenere ciphers [29], data privacy protection [30], image segmentation [31], spam detection [32], the design of multidocument summarization extractor [33], and robot path planning [34, 35].

## 4. Cuckoo Algorithm Based on Global Feedback

The cuckoo algorithm based on global feedback is described in the following sections.

### 4.1. Motivation

In Lévy flights, it is difficult to balance the large-scale global exploration in the early stages and the local fine-grained search in the later stages with a fixed step size and probability of nest discovery. To improve the dynamic search characteristics of the CS algorithm, an adaptive adjustment scheme of step size *α* and the probability of nest discovery *Pa* are proposed to coordinate the overall search performance of the algorithm according to the evolution of the globally optimal individuals. Due to the guidance of the optimal solution in the population, the CS algorithm is easy to converge prematurely when solving some complex optimization problems and falls into the local optimal value prematurely. Even at the end of the whole algorithm, the algorithm fails to jump out of the local optimum. Given this situation, we introduce additional dynamic parameters to adjust the step size and *Pa* according to the change of the best fitness value of the current generation. If it has been determined that the algorithm is stuck in a local optimum after many rounds, then we consider resetting the algorithm.

### 4.2. Step Size and **Pa** Adjustment

The main parameters in the CS algorithm are step size *α* and the probability of the bird's nest being found *Pa*. *α* mainly controls the step size of the algorithm, and the value of *Pa* mainly controls the diversity of the algorithm for each round of exploration. If a smaller value of *Pa* is used, the global bird's nest will tend to be concentrated, and the local search ability of the algorithm will be strengthened, but the diversity of the algorithm gets worse. If the value of *Pa* is small but the value of *α* is large, the performance of the algorithm will be poor, resulting in a large increase in the number of iterations. If the value of *Pa* is large but the value of *α* is small, the convergence rate is fast, but the optimal solution may not be found. In the original CS algorithm, both *Pa* and *α* use fixed values, which cannot be changed during the algorithm iteration. It is difficult for the algorithm to guarantee the speed of global exploration in the early stage, and the lack of overall exploration capability may contribute to the inability of the algorithm to explore the global optimum. And in the later stage, due to the slightly larger step size, it is difficult for the algorithm to perform a very fine search locally. To improve the performance of the algorithm, an adaptive step size formula is introduced as follows:

(8) $$\begin{array}{c} \alpha \left(t\right)={m*\alpha }_{\max}\exp\left(c\right)\text{,} \end{array}$$

where

(9) $$\begin{array}{c} c=\ln\left(\frac{\alpha_{\min}}{\alpha_{\max}}\right)*\frac{t}{MaxIt}\text{,} \end{array}$$

and the formula for *Pa* is as follows:

(10) $$\begin{array}{c} P_{a}\left(t\right)=\frac{m*2{Pa}_{\max}}{\left(1+\exp\;\left(T*t/MaxIt\right)\right)}\text{,} \end{array}$$

where *t* represents the current number of iterations, MaxIt represents the total number of iterations, *α*_min_ and *α*_max_ represent the preset minimum step size and the preset maximum step size, respectively, *Pa*_max_ represents the preset maximum value of *Pa*, *T* represents a constant between 1 and 10, and *m* represents a dynamically adjusted global parameter, which will be introduced in the following sections.

Equations (8) and (9) refer to parts of [9], on which we change the values of the upper and lower bounds of their definitions and use dynamic global variables to adjust their values again. So, we choose $\begin{array}{c} \alpha_{\max}=0.5\;and\alpha_{\min}=0.005 \end{array}$.

For equation (10), inspired by the sigmoid function [36] in deep learning, we hope the value of parameter *Pa* to smoothly over from a larger value in the early stage to a smaller value in the later stage like the inverted sigmoid function during the whole algorithm, so equation (10) is designed. In order to prevent the curve from changing too drastically or slowly, an adjustable variable *T* is added on top of it to control the whole change process.

To ensure that *P*_*a*_(*t*) can obtain a large value when *t* is small at the beginning of the algorithm, we choose *Pa*_max_=0.5. To ensure that the curve of *P*_*a*_(*t*) changes quickly but not too steeply, we did experiments on the value of *T*. The experimental results show that the overall effect is best when *T* is taken as 6, so we choose $\begin{array}{c} T=6 \end{array}$.

### 4.3. Global Feedback and Re-Fly

The previous formulas of *Pa* and *α* ensure that *Pa* and *α* can be quickly changed from larger values in the previous stage to relatively small values as the algorithm progresses. This process is essentially irreversible, and the algorithm is likely to fall into a local optimum, thus affecting the subsequent global exploration. For this, we introduce a dynamic adjustment parameter *m* and set the initial value of *m* to 1. As the algorithm runs, the formula for *m* is as follows:

(11) $$\begin{array}{c} m=\left\{\begin{array}{cc} 1\text{,} & f_{best}^{new}<f_{best}\text{,}\\ m+0.001\text{,} & otherwise\text{.} \end{array}\right. \end{array}$$

If the optimal fitness value does not change after this round of iteration, we will increase the value of *m*. Due to the influence of *m*, the step size *α* and *Pa* of the algorithm are enlarged, and the global exploration ability of the algorithm is improved. If the optimal fitness value of the algorithm changes after a certain round of iteration, we consider that the algorithm has found a better solution and reset *m* to 1 at this point. If the algorithm falls into a local optimal value (nonglobal optimal value), as *m* continues to increase, it still does not obtain a better optimal fitness value. When *m* > 2, we will make a judgment at this time.

If *t* < MaxIt/2, we will keep the current best fitness value, reset all nests, set *m* to 1, and re-execute the algorithm. This stage is called “re-fly.” Otherwise, we will continue to execute the algorithm and expand *m*, making the step size gradually expand until a better fitness value appears.

In the traditional CS algorithm and some improved versions of the CS algorithm, the algorithm may converge prematurely at the beginning of the algorithm and fall into a local optimum, and at the end of the entire algorithm, the algorithm does not obtain a better value. Therefore, the “re-fly” method is introduced in GFCS. If the best fitness value does not change after 1000 iterations and the current number of rounds does not reach half of the total number of rounds, the subsequent computing power is reserved to obtain better results.

In terms of the previous descriptions, the implementation of GFCS is shown in Algorithm 2.

**Figure 1:**
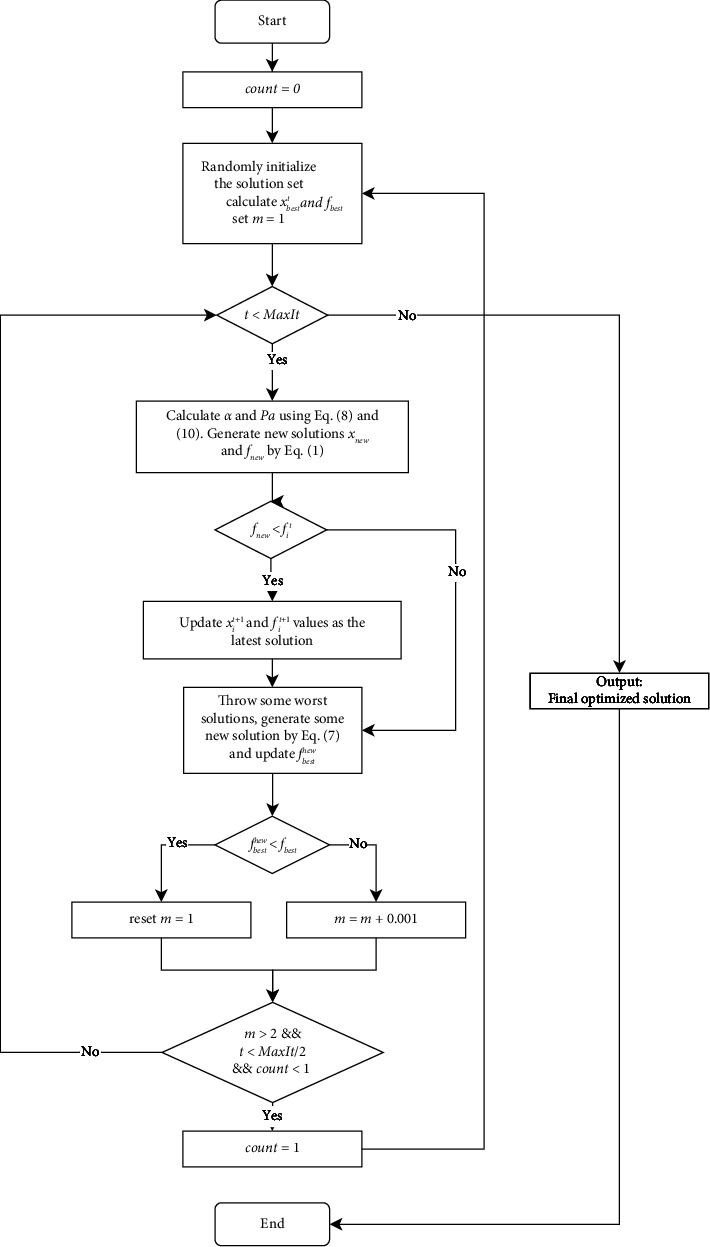
The flowchart of the GFCS algorithm.

To show the algorithm process more visually, the flowchart of the algorithm is shown in Figure 1.

### 4.4. Algorithm Complexity Analysis

To demonstrate that the GFCS algorithm does not increase the time and space complexity of the CS algorithm, we analyse the algorithmic complexity of GFCS and CS. For the CS algorithm, assuming that the dimension of the problem is *D*, the time to evaluate the *D*-dimensional function is positively related to *D*, the total number of iterations is *G*, and the population size is *n*. Therefore, the time complexity of the CS algorithm is approximately: *O*(GDn).

GFCS is improved only in the Lévy flight phase and is consistent with the CS algorithm in the local walk phase, so we only need to analyse the differences in the previous phase. GFCS requires additional calculations of variables *α* and *Pa* in each iteration, determines and updates the value of the parameter *m*. The time consumption during the calculation is only related to the parameters *G* and not to *D* and *n*. In addition, “re-fly” is performed at most once in the algorithm, and the time consumption is only related to *n* and *D* and not to *G*. Therefore, the total time complexity of GFCS is still *O*(GDn).

In terms of space complexity, GFCS does not use extra storage space to store data, so it does not increase the space complexity of the algorithm. In summary, the complexity of GFCS is in the same order of magnitude as that of the original CS. In the subsequent experiments, we will further compare the total time consumption of GFCS with the original CS on the test set functions.

## 5. Experimental Study

The experimental study is described in the following sections.

### 5.1. Experimental Environment and Benchmark Functions

To verify the performance of GFCS, experiments are carried out on the test set of CEC2013 [37], which is widely used internationally. CEC2013 contains 28 test functions, among which *f*1–*f*5 are unimodal functions, *f*6–*f*20 are multimodal functions, and *f*21–*f*28 are combination functions. The solution constraints of the functions are in the range of [−100,100]. All experiments were performed on the Windows 10 platform, and all algorithms were implemented in MATLAB R2021a.

In our experiments, *f*_opt_ represents the standard optimal value of the objective function and *f*_min_ represents the actual optimal value of the objective function obtained by our algorithm. We record the following equation as the criterion for the algorithm detection:

(12) $$\begin{array}{c} res=f_{\min}-f_{opt}\text{.} \end{array}$$

Therefore, the closer res is to 0, the better it is. Furthermore, to reduce the statistical error, the average error of all these independently running functions was chosen as the performance metric. To ensure the fairness of the experiment, the population size *n* is set to 25, with the dimension *D*=30 and the maximum number of iterations MaxIt=20000. Each test function was run 30 times in the same environment, and its mean and standard error values were recorded.

### 5.2. Comparison with CS and Other Variants

To explore the accuracy and convergence of GFCS, a comparative analysis was carried out with the original CS and its seven variants. The seven CS variants were CS [6], ACS [10], NACS [20], GCS [17], ICS [9], ACSA [11], VCS [12], and MSRCS [23].  Table 1 lists the core ideas and specific parameters of each algorithm.

In this section, we compare GFCS with the original CS and 7 improved CS variants. Tables 2 and 3 show the 30-dimensional test results of GFCS and other 8 CS algorithms in the CEC2013 test set. In the tables, bold letters indicate the best results, “Mean” and “Std” represent the mean and standard error values, respectively. In addition, the average ranking results of the Friedman test are added at the bottom of the table, where “+” indicates that the results are better than the algorithm, “−” indicates that are worse, and “≈” indicates that the results are not much different.

The data in Tables 2 and 3 show that GFCS works best on *f*2, *f*4, *f*6, *f*7, *f*9, *f*12, *f*13, *f*15, *f*18, *f*19, *f*20, *f*23, *f*24, *f*25, *f*26, and *f*27, which include unimodal functions, multimodal functions, and combined functions. In unimodal functions, the effect of GFCS is clearly better than other algorithms on *f*2 and *f*4, and the performance of each algorithm tends to be consistent on *f*1, *f*3, and *f*5. The results of GFCS on multimodal functions *f*6, *f*7, *f*9, *f*12, *f*13, *f*15, *f*18, *f*19, and *f*20 are all better than other algorithms, and the results of *f*17 are better than other algorithms except VCS. And in the combined functions *f*23, *f*24, *f*25, *f*26, and *f*27, the results of GFCS are better, as it obtains better optimal values. Through the results of the Friedman test at the bottom of Tables 2 and 3, it can be found that GFCS beats other algorithms on at least 18 functions compared to other algorithms. And the average ranking of GFCS for the 28 tested functions in the two tables are 1.43 and 1.46, respectively, which ranks first compared to the other 8 CS algorithms. The analysis shows that GFCS has good stability and convergence for different types of problems.

**Figure 2:**
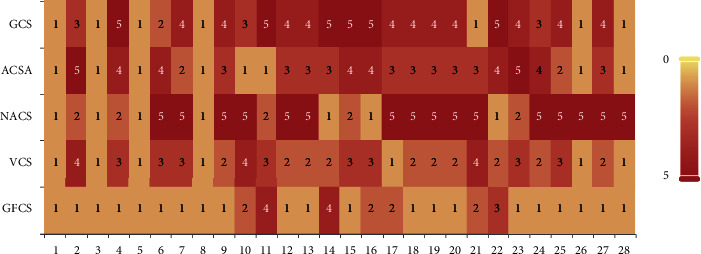
System-stacked histogram of ranking for GCS, ACSA, NACS, VCS, and GFCS on CEC2013 (*D* = 30).

**Figure 3:**
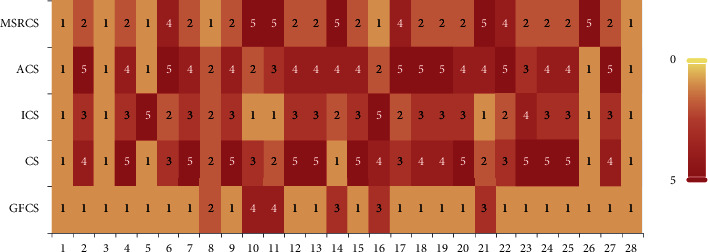
System-stacked histogram of ranking for MSRCS, ACS, ICS, CS, and GFCS on CEC2013 (*D* = 30).

In addition, to visually display the ranking of the results in Tables 2 and 3, the ranking of the average error value (minimization problem) of each function is summarized, and stacked histograms based on the ranking statistics are drawn. Figures 2 and 3 shows that each ranking is represented by a color block. The better the algorithm performs, the lighter the color of the corresponding block is.  Figure 2 shows the ranking of GCS, ACSA, NACS, VCS, and GFCS, and Figure 3 shows the ranking of MSRCS, ACS, ICS, CS, and GFCS.

As can be seen from the figures, the white block that marks the first has the largest proportion in GFCS, and GFCS does not obtain the red square that marks the fifth, indicating that GFCS has the best performance compared to other algorithms. In addition, on some functions, such as *f*11 and *f*16, GFCS does not achieve the first place, but still locates in the second or third position, showing that GFCS is still competitive. Overall, for the CEC2013 test set with *D* = 30, GFCS has a considerable advantage over other algorithms.

**Figure 4:**
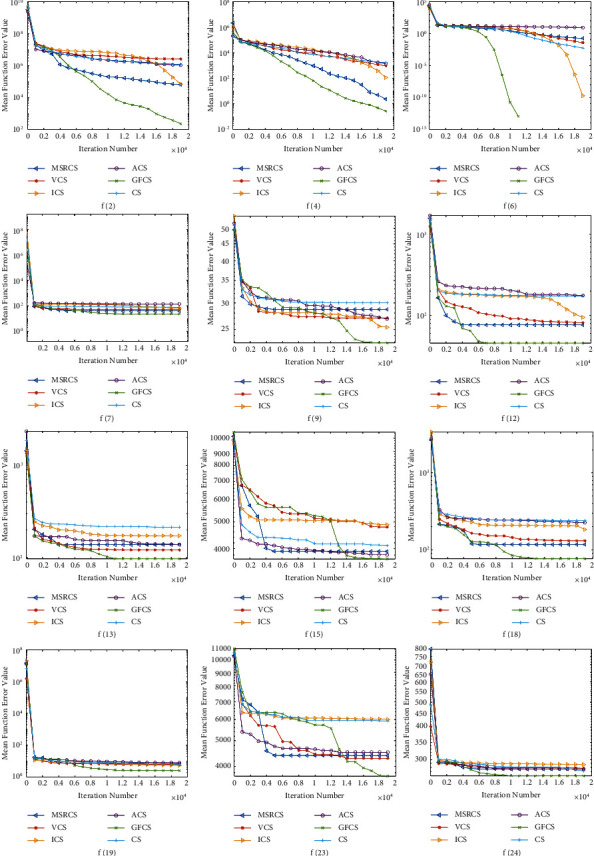
Convergence curves of MSRCS, VCS, ICS, ACS, CS, and GFCS on part functions of CEC2013 (*D* = 30).

To further illustrate the convergence capability and optimization accuracy of GFCS, the original CS and four representative CS variants are selected in conjunction with the comparison results above. In addition, we select representative functions from the unimodal, multimodal, and combinatorial functions of the CEC2013 test set. They are *f*2, *f*4, *f*6, *f*7, *f*9, *f*12, *f*13, *f*15, *f*18, *f*19, *f*23, and *f*24, where *f*2 and *f*4 are unimodal functions, *f*6, *f*7, *f*9, *f*12, *f*13, *f*15, *f*18, and *f*19 are multimodal functions, and *f*23 and *f*24 are combined functions. Based on the above algorithms and test functions, their convergence curves are plotted in Figure 4, where the horizontal axis indicates the number of iterations and the vertical axis indicates the error values.

From Figure 4, it can be observed that GFCS converges significantly faster than other competitors on *f*2, *f*4, *f*6, *f*12, *f*19, and *f*24. For the remaining functions, although the convergence speed of GFCS is not the fastest compared with other algorithms, the best optimization results can still be achieved as the iteration progresses.

In addition, we can find that, for functions *f*2, *f*4, *f*6, *f*13, *f*19, and *f*24, GFCS can obtain a smoother descending curve during the convergence process because GFCS can quickly adjust the parameters Pa and step size *α*, to obtain faster convergence speed and more precise search results.

For other multimodal functions or mixed functions, these functions tend to make the algorithm fall into a local optimum. After the algorithm falls into the local optimum in a very early period, its optimum value is usually difficult to change greatly. For these functions, the advantages of GFCS are even more obvious. As *f*9, *f*12, *f*15, *f*18, and *f*23, Figure 4 shows that the optimization curve of GFCS falls rapidly again after a period of stagnation, jumping out of the current local optimum. At this time, the global feedback part and “re-fly” of GFCS play a role, making the algorithm jump out of the local optimum and find a better solution. Since the algorithm balances exploration and exploitation according to the optimization of each round of iterations, the convergence curve is not a continuous decline but presents a state of gradual optimization in stages to approach the global optimal solution.

According to the previous analysis, GFCS has a good convergence speed and optimization ability on various types of test functions and is able to jump out of local extremes. Therefore, we can conclude that GFCS achieves better performance than other algorithms when dealing with 30-dimensional problems.

### 5.3. Effect of Dimension Growth

**Figure 5:**
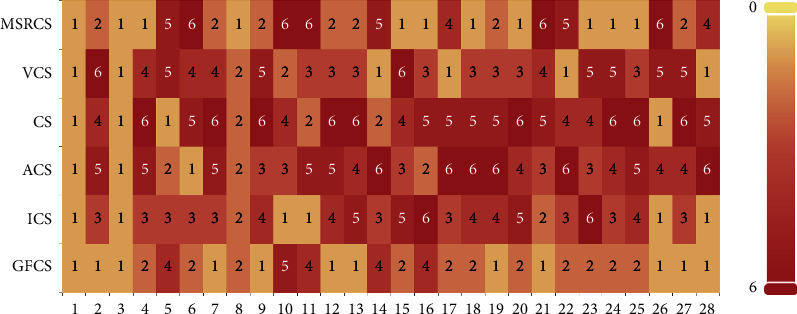
System-stacked histogram of ranking for MSRCS, VCS, CS, ACS, ICS, and GFCS on CEC2013 (*D* = 50).

As can be seen in the above sections, GFCS outperforms others in handling the 30-dimensional functions on the CEC2013 benchmark features. However, for a good algorithm, it should also be able to generate high-quality solutions to high-dimensional problems. To study the impact of dimensional growth on GFCS performance, we investigate the scalability of the algorithm on 28 test functions of CEC2013 with problem dimension size scaled from 30 to 50 In this section, we choose MSRCS, VCS, ACS, ECS, ICS, and GFCS, which performed better in the previous sections for comparison experiments, and the experimental results are shown in Table 4 and Figure 5.

From Table 4, GFCS wins on *f*2, *f*7, *f*9, *f*12, *f*13, *f*19, *f*21, and *f*27. Although it does not finish first on many other functions, it still achieves a relatively high ranking. Likewise, MSRCS is the champion on *f*4, *f*8, *f*15, *f*16, *f*18, *f*20, *f*23, *f*24, and *f*25. ICS is the *f*10 and *f*11 champion. VCS obtains the best results on *f*14, *f*17, and *f*22, CS obtains the best solution on *f*5, and ACS obtains the best solution on *f*6. Furthermore, all algorithms achieve the same result on *f*3. As can be seen at the bottom of the table, compared with other algorithms, GFCS outperforms at least 17 functions and has an average rank of 1.96. In addition, Figure 5 shows that GFCS still has the lightest overall color block and high rankings on most functions. More specifically, GFCS achieves the first- or second-best results on most functions. It still has a clear advantage over other algorithms. Based on all previous experimental analyses, we can conclude that although the advantage of GFCS mildly decreases when the dimensionality of the problem increases from 30 to 50 dimensions, GFCS is still the best algorithm to handle these benchmark functions combining the results of the previous experiments.

**Figure 6:**
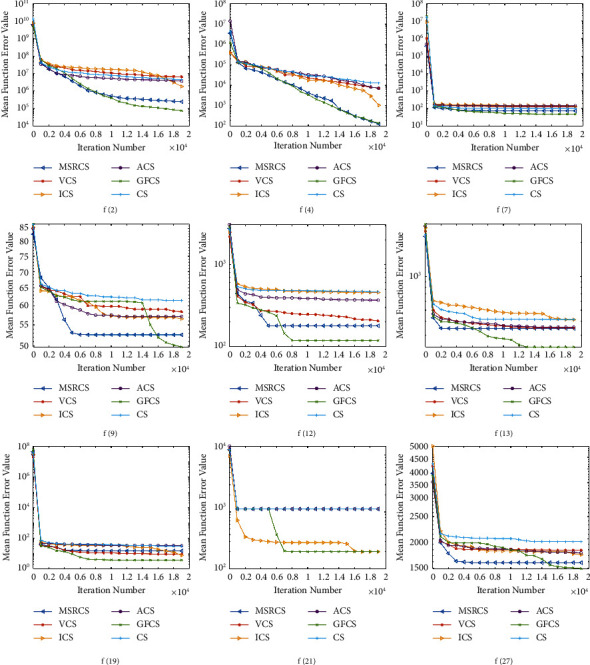
Convergence curves of MSRCS, VCS, ICS, ACS, CS, and GFCS on part functions of CEC2013 (*D* = 50).

To visually compare the optimization of GFCS with other algorithms in the case of *D* *=* 50, we draw the optimization images of some functions in Figure 6. Figure 6 shows that, on the six functions *f*2, *f*7, *f*12, *f*13, *f*19, and *f*21, GFCS is significantly faster than the other competitors and is able to achieve better optimal values. On *f*4, *f*9, and *f*27, although GFCS is not significantly faster than the other competitors, it is relatively fast and can eventually achieve the best fitness value. In conclusion, the proposed GFCS has a better performance compared to the other competitors.

### 5.4. Comparison with Other Evolutionary Algorithms

To further confirm the superiority of GFCS, we select some other evolutionary algorithms for comparison. The differential evolution algorithm [2] (DE) and firefly algorithm [5] (FA) are widely studied and used swarm intelligence optimization algorithms. To further verify the performance of GFCS, DE, FA, and some variants of DE, ABC, and BSO are selected for comparison, the variants being NABC [38], ABCX [39], CUDE [40], and MSBSO [41].

In view of the fairness of the experiments, the population size = 25, the problem dimension = 30, and the number of evaluations = 1*E*6 are set for these algorithms, and each test function independently runs 30 times. For some other parameters of the NABC, ABCX, CUDE, and MSBSO, we follow the settings in the literature, and the parameters of GFCS are consistent with the previous tests. The experimental results are shown in Table 5, and the best result is shown in **bold**.

According to Table 5, GFCS finds the best values on *f*1, *f*2, *f*5, *f*6, *f*10, *f*12, and *f*26. Likewise, NABC behaves well on *f*11, *f*15, *f*16, *f*19, *f*25, and *f*26 and *F*A provides the best solutions on *f*8 and *f*9, while does not yield optimal results for any function. In addition, ABCX performs best on *f*14, *f*17, *f*21, *f*22, *f*23, *f*27, and *f*28, CUDE finds the best values on *f*3 and *f*4, and MSBSO provides the best solutions on *f*1, *f*5, *f*7, *f*12, *f*13, *f*18, *f*20, and *f*24. In terms of average ranking results, GFCS generates an average rank value of 3.00, and ranks first, followed by the MSBSO algorithm with a ranking of 3.03, CUDE with a ranking of 3.43, NABC with a ranking of 3.71, and ABCX with a ranking of 4.25, respectively. The results of the average rank obtained by the Friedman test show that GFCS is still competitive with other swarm intelligence algorithms. In addition, it can be seen from the bottom of Table 5 that, compared with these algorithms, GFCS surpasses the other algorithms in most functions, which show that GFCS still has a relatively large advantage compared with other evolutionary algorithms.

**Figure 7:**
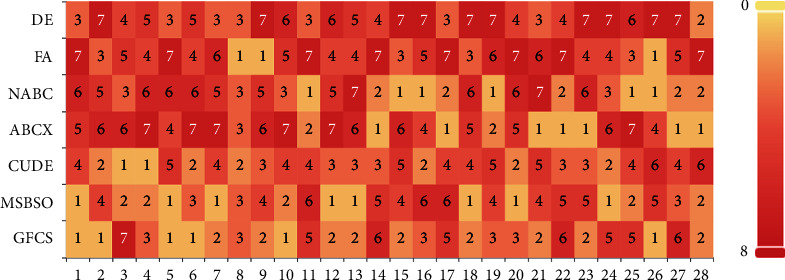
System-stacked histogram of ranking for DE, FA, NABC, ABCX, CUDE, MSBSO, and GFCS on CEC2013 (*D* = 30).

Figure 7 shows that we plotted a superimposed histogram based on the ranking statistics to better visualize the ranking of the results in Table 5. Figure 7 shows that GFCS has lighter overall color block rankings and ranks in the top three on 19 functions. Except for the eighth ranking of GFCS on function 3, GFCS does not achieve any other sixth or seventh ranking. By comparing the ranking with other algorithms, we can see that GFCS is still competitive.

To further verify the performance of GFCS, we select some other classical evolutionary algorithms for comparison. The genetic algorithm [42], GA, particle swarm optimization [3] (PSO), ant colony algorithm [43] (ACO), artificial bee colony algorithm [4] (ABC), and brain storm optimization algorithm [44] (BSO) are selected for comparison.

In view of the fairness of the experiments, the population size *N* = 25, the problem dimension *D* = 30, and the number of evaluations = 1*E*6 are set for these algorithms, and each test function independently runs 30 times. For GA, the probability of crossover is set to 1, and the probability of mutation is set to 0.01. For PSO, we set the personal learning coefficient = 1.5 and the global learning coefficient = 2.0. For ACO, the evaporation rate of pheromone is set to 0.1. Moreover, for ABC, the parameter limit is set to (0.6*∗*N)*∗D*. For BSO, the number of clusters is set to five. For other parameters of the GA, PSO, ACO, ABC, and BSO, we followed the settings in the literature, and the parameters of GFCS are consistent with the previous tests. The experimental results are shown in Table 5, and the best result is shown in bold.

According to Table 6, GFCS finds the best values on all functions except *f*3, *f*8, *f*10, *f*15, and *f*16. In terms of the average ranking results, GFCS produces an average ranking value of 1.39, which has a greater advantage over other SI algorithms. In addition, it can be seen from the bottom of Table 6 that GFCS beats these classical algorithms on most functions, which shows that the GFCS algorithm has a greater advantage over them.

**Figure 8:**
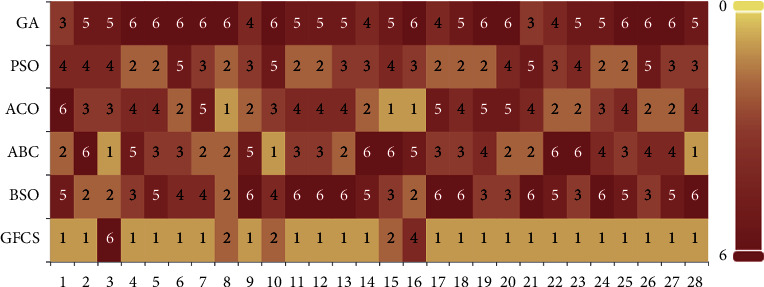
System-stacked histogram of ranking for GA, PSO, ACO, ABC, BSO, and GFCS on CEC2013 (*D* = 30).

We draw a stacked histogram based on the ranking statistics to better visualize the ranking of the results in Table 6, as detailed in Figure 8. Figure 8 shows that GFCS has lighter overall color blocks and ranks first on 23 functions. Moreover, GFCS does not achieve any other fifth or sixth ranking except the sixth ranking on function 3. By comparing the rankings with these SI algorithms, we can conclude that GFCS has a clear superiority in searching for the global optimum.

### 5.5. Comparison of Calculation Time

To demonstrate the effectiveness of GFCS in terms of running time, we calculate the time of running the 28 test functions of the CEC2013 test set with GFCS and the original CS algorithm in different dimensions. Among them, the dimensions are set to 30 and 50. For the other parameters, the upper limit of the number of iterations is set to 20,000, and the population size is set to 25. To exclude measurement chance, each function on the CEC2013 test set runs 10 times independently, and the total run time is calculated. The experimental results are shown in Table 7.

Table 7 shows that there is no significant difference in runtime between CS and GFCS for either dimension *D* = 30 or *D* = 50. This again validates the complexity analysis of CS and GFCS in Section 4.4, where there is no significant difference between GFCS and CS in terms of time complexity.

## 6. Conclusion

This article proposes a global feedback-based cuckoo search algorithm (GFCS). In GFCS, we introduce the concept of global feedback and the “re-fly” mechanism. In addition, we set new parameter formulas that change with the number of iteration rounds and are controlled by the dynamic global variables. To evaluate the performance of the GFCS algorithm, GFCS is compared with the other eight variants of the CS algorithm and several classical evolutionary algorithms and their variants. Based on the experimental results, the following conclusions can be drawn:

- GFCS algorithm adopts a global feedback strategy and the “re-fly” mechanism in the optimization search process. According to the evolution of the current generation, GFCS adjusts the parameters of the algorithm globally during the evolution process, effectively accelerates the algorithm's convergence speed, and enriches the population and the diversity of learning.
- Compared with CS, some CS variants, and several SI algorithms in the experiment, the GFCS algorithm has faster convergence speed and better convergence accuracy.
- As we compare in Sections 4.4 and 5.5, the time and space complexity of GFCS is comparable to that of the traditional CS algorithm, which means that the GFCS algorithm does not improve the complexity of the algorithm.

In the future, we intend to extend our current work in the following directions. Firstly, for the switching parameters, we will try to adjust the adaptive adjustment mechanism or introduce a multistrategy mechanism to further improve the search ability. Secondly, we will consider the application of GFCS to some other scientific problems, such as applying the algorithm to the field of machine learning or deep learning. Thirdly, we will discuss the application of the algorithm to some practical problems.

## Figures and Tables

**Algorithm 1:**
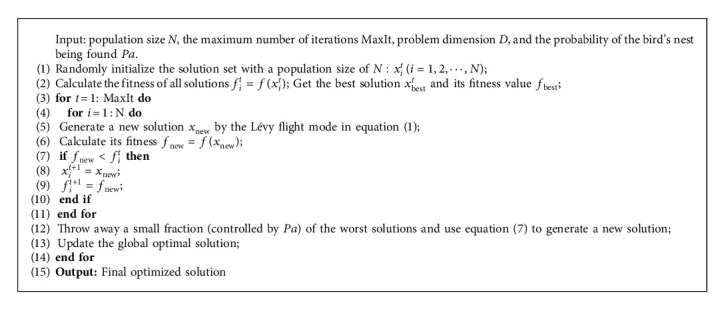
Cuckoo search (CS) original algorithm.

**Algorithm 2:**
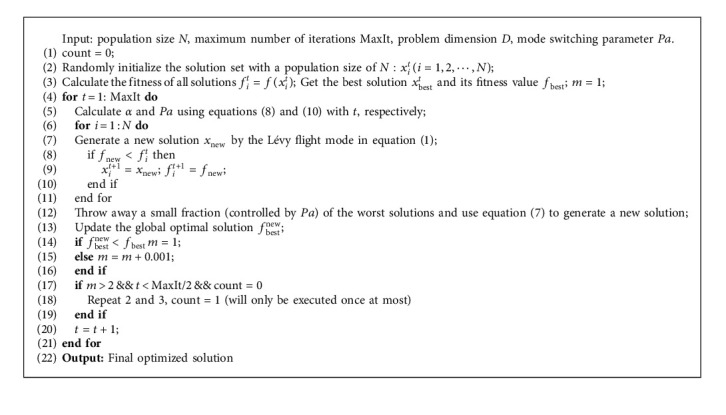
Cuckoo search based on the global feedback.

**Table 1:** Core ideas and parameter settings for CSs.

Algorithms	Core ideas	Parameter settings
CS	Basic CS	*α*=0.01 and *P*_*a*_=0.25
ACS	The Lévy flight is abandoned and a new step control formula and iteration formula are introduced	*P*_*a*_=0.25
NACS	Neighborhood attraction is introduced to improve the performance of CS	*Pa*=0.25, *m*=3, and *Ps*=0.8
ICS	Control the step size and *Pa* with the number of iterations to improve the performance of CS	$\begin{array}{c} \alpha_{\max}=0.5\;and\alpha_{\min}=0.01 \end{array}\begin{array}{c} P_{a\max}=0.5\;andP_{a\min}=0.005 \end{array}$
ACSA	Compare the current fitness value with the average fitness value to decide on the two different step size methods	*α*_*U*_=0.8 and *α*_*L*_=0.2
VCS	In VCS, a varied scale factor is added to the step adjustment of the Lévy flight	*β*=1.5 and*P*_*a*_=0.25
GCS	Using Gaussian distribution instead of the Lévy flight to improve the algorithm performance	*μ*=0.0001, *σ*_0_=0.5, *and* *P*_*a*_=1.5
MSRCS	Based on reconciliatory strategy to update solutions, MSRCS tries to strike a balance between exploration and exploitation	$\begin{array}{c} p_{a}=0.25\text{,} \end{array}\begin{array}{c} p_{1}=0.8,{and\;p}_{2}=0.5 \end{array}$
GFCS	Dynamic variables and “re-fly” mechanism are added to the operation of the algorithm, and feedback is obtained on a per-round iteration to improve algorithm performance	$\begin{array}{c} \alpha_{\max}=0.5,\alpha_{\min}=0.005 \end{array}\begin{array}{c} {Pa}_{\max}=0.5,and\;T=6 \end{array}$

**Table 2:** The test result of GCS, ASCA, NACS, VCS, and GFCS on CEC2013 (*D* = 30).

Function	Mean/std	GCS	ASCS	NACS	VCS	GFCS
***F*1**	Mean	**0.00*E*** + **00**	**0.00*E*** + **00**	**0.00*E*** + **00**	**0.00*E*** + **00**	**0.00*E*** + **00**
	Std	**0.00*E*** + **00**	**0.00*E*** + **00**	**0.00*E*** + **00**	**0.00*E*** + **00**	**0.00*E*** + **00**

***F*2**	Mean	9.47*E* + 05	2.22*E* + 06	1.41*E* + 05	1.44*E* + 06	**1.96*E*** + **03**
	Std	3.22*E* + 05	9.51*E* + 05	6.01*E* + 04	5.45*E* + 05	**2.48*E*** + **03**

***F*3**	Mean	**1.00*E*** + **10**	**1.00*E*** + **10**	**1.00*E*** + **10**	**1.00*E*** + **10**	**1.00*E*** + **10**
	Std	**0.00*E*** + **00**	**0.00*E*** + **00**	**0.00*E*** + **00**	**0.00*E*** + **00**	**0.00*E*** + **00**

***F*4**	Mean	4.15*E* + 03	8.89*E* + 02	1.12*E* + 00	7.49*E* + 02	**3.97*E***−**01**
	Std	1.46*E* + 03	2.88*E* + 02	**4.04*E*** − **01**	2.51*E* + 02	4.98*E* − 01

***F*5**	Mean	**0.00*E*** + **00**	**0.00*E*** + **00**	**0.00*E*** + **00**	**0.00*E*** + **00**	**0.00*E*** + **00**
	Std	**0.00*E*** + **00**	**0.00*E*** + **00**	**0.00*E*** + **00**	**0.00*E*** + **00**	**0.00*E*** + **00**

***F*6**	Mean	1.52*E* + 00	3.74*E* + 00	1.33*E* + 01	2.33*E* + 00	**1.36*E*** − **13**
	Std	2.89*E* + 00	8.59*E* + 00	1.51*E* + 01	6.58*E* + 00	**4.37*E*** − **13**

***F*7**	Mean	6.80*E* + 01	5.22*E* + 01	1.39*E* + 02	5.77*E* + 01	**3.53*E*** + **01**
	Std	3.03*E* + 01	2.60*E* + 01	3.50*E* + 01	1.39*E* + 01	**7.09*E*** + **00**

***F*8**	Mean	**2.09*E*** + **01**	**2.09*E*** + **01**	**2.09*E*** + **01**	**2.09*E*** + **01**	**2.09*E*** + **01**
	Std	**2.40*E*** − **02**	5.16*E* − 02	3.27*E* − 02	3.19*E* − 02	5.64*E* − 02

***F*9**	Mean	2.91*E* + 01	2.86*E* + 01	3.14*E* + 01	2.73*E* + 01	**2.16*E*** + **01**
	Std	**9.52*E*** − **01**	2.60*E* + 00	2.54*E* + 00	1.82*E* + 00	2.68*E* + 00

***F*10**	Mean	1.74*E* − 02	**1.47*E*** − **02**	1.44*E* − 01	2.02*E* − 02	1.63*E* − 02
	Std	2.01*E* − 02	**1.48*E*** − **02**	8.60*E* − 02	1.87*E* − 02	1.49*E* − 02

***F*11**	Mean	1.91*E* + 01	**4.03*E*** + **00**	6.91*E* + 00	8.51*E* + 00	1.21*E* + 01
	Std	1.17*E* + 01	**2.08*E*** + **00**	3.97*E* + 00	3.97*E* + 00	3.20*E* + 00

***F*12**	Mean	9.73*E* + 01	9.08*E* + 01	3.07*E* + 02	7.66*E* + 01	**5.38*E*** + **01**
	Std	1.76*E* + 01	1.26*E* + 01	9.22*E* + 01	1.17*E* + 01	**1.12*E*** + **01**

***F*13**	Mean	1.48*E* + 02	1.37*E* + 02	3.33*E* + 02	1.19*E* + 02	**1.00*E*** + **02**
	Std	**1.28*E*** + **01**	2.09*E* + 01	5.26*E* + 01	1.99*E* + 01	2.62*E* + 01

***F*14**	Mean	1.18*E* + 03	9.85*E* + 02	**7.07*E*** + **02**	8.30*E* + 02	1.02*E* + 03
	Std	3.68*E* + 02	3.49*E* + 02	2.73*E* + 02	4.00*E* + 02	**2.68*E*** + **02**

***F*15**	Mean	4.59*E* + 03	4.41*E* + 03	3.65*E* + 03	4.27*E* + 03	**3.46*E*** + **03**
	Std	5.07*E* + 02	**3.29*E*** + **02**	3.98*E* + 02	3.47*E* + 02	7.47*E* + 02

***F*16**	Mean	1.60*E* + 00	1.46*E* + 00	**5.04*E*** − **01**	1.42*E* + 00	1.38*E* + 00
	Std	1.20*E* − 02	1.78*E* − 01	**9.83*E*** − **02**	2.37*E* − 01	3.41*E* − 01

***F*17**	Mean	7.53*E* + 01	5.35*E* + 01	1.49*E* + 02	**4.70*E*** + **01**	5.29*E* + 01
	Std	9.63*E* + 00	9.61*E* + 00	5.27*E* + 01	**7.15*E*** + **00**	7.81*E* + 00

***F*18**	Mean	1.59*E* + 02	1.45*E* + 02	3.63*E*+02	1.32*E*+02	**8.25*E*** + **01**
	Std	**8.17*E*** + **00**	1.59*E* + 01	9.91*E* + 01	9.88*E* + 00	1.89*E* + 01

***F*19**	Mean	8.20*E* + 00	5.95*E* + 00	1.75*E* + 01	4.34*E* + 00	**2.29*E*** + **00**
	Std	1.44*E* + 00	1.85*E* + 00	5.57*E* + 00	1.45*E* + 00	**4.77*E*** − **01**

***F*20**	Mean	1.21*E* + 01	1.19*E* + 01	1.33*E* + 01	1.19*E* + 01	**1.11*E*** + **01**
	Std	**2.94*E*** − **01**	3.75*E* − 01	8.24*E* − 01	3.65*E* − 01	4.84*E* − 01

***F*21**	Mean	2.48*E* + 02	2.56*E* + 02	3.25*E* + 02	2.63*E* + 02	**2.47*E*** + **02**
	Std	**4.62*E*** + **01**	6.35*E* + 01	1.03*E* + 02	5.64*E* + 01	5.16*E* + 01

***F*22**	Mean	1.94*E* + 03	1.39*E* + 03	6.07*E* + 02	9.87*E* + 02	1.04*E* + 03
	Std	5.21*E* + 02	5.55*E* + 02	**2.66*E*** + **02**	5.76*E* + 02	3.16*E* + 02

***F*23**	Mean	5.04*E* + 03	5.08*E* + 03	4.75*E* + 03	4.77*E* + 03	**3.68*E*** + **03**
	Std	5.96*E* + 02	3.70*E* + 02	4.20*E* + 02	**3.00*E*** + **02**	5.32*E* + 02

***F*24**	Mean	2.75*E* + 02	2.75*E* + 02	2.91*E* + 02	2.73*E* + 02	**2.59*E*** + **02**
	Std	7.36*E* + 00	**6.57*E*** + **00**	8.92*E* + 00	1.05*E* + 01	7.22*E* + 00

***F*25**	Mean	2.90*E* + 02	2.84*E* + 02	3.06*E* + 02	2.85*E* + 02	**2.75*E*** + **02**
	Std	**3.32*E*** + **00**	9.33*E* + 00	9.78*E* + 00	6.18*E* + 00	6.45*E* + 00

***F*26**	Mean	**2.00*E*** + **02**	**2.00*E*** + **02**	2.54*E* + 02	**2.00*E*** + **02**	**2.00*E*** + **02**
	Std	1.74*E* − 02	3.31*E* − 02	8.41*E* + 01	2.32*E* − 02	**1.57*E*** − **04**

***F*27**	Mean	1.11*E* + 03	1.03*E* + 03	1.17*E* + 03	1.02*E* + 03	**8.57*E*** + **02**
	Std	**2.58*E*** + **01**	1.51*E* + 02	6.59*E* + 01	3.50*E* + 01	1.42*E* + 02

***F*28**	Mean	**3.00*E*** + **02**	**3.00*E*** + **02**	1.90*E* + 03	**3.00*E*** + **02**	**3.00*E*** + **02**
	Std	**0.00*E*** + **00**	**0.00*E*** + **00**	1.33*E* + 03	**0.00*E*** + **00**	**0.00*E*** + **00**

**Average ranking**		2.71	2.79	3.46	2.25	1.43
+/−/≈		21/1/6	19/4/5	20/4/4	18/4/6	

In the table, bold letters indicate the best results, “Mean” and “Std” represent the mean and standard error values, respectively.

**Table 3:** The test result of CEC2013 of MSRCS, ACS, ICS, CS, and GFCS on CEC2013 (*D* = 30).

Function	Mean/std	MSRCS	ACS	ICS	CS	GFCS
***F*1**	Mean	**0.00*E*** + **00**	**0.00*E*** + **00**	**0.00*E*** + **00**	**0.00*E*** + **00**	**0.00*E*** + **00**
	Std	**0.00*E*** + **00**	**0.00*E*** + **00**	**0.00*E*** + **00**	**0.00*E*** + **00**	**0.00*E*** + **00**

***F*2**	Mean	3.78*E* + 04	1.06*E* + 06	6.65*E* + 04	8.91*E* + 05	**1.96*E*** + **03**
	Std	1.59*E* + 04	3.74*E* + 05	4.40*E* + 04	3.76*E* + 05	**2.48*E*** + **03**

***F*3**	Mean	**1.00*E*** + **10**	**1.00*E*** + **10**	**1.00*E*** + **10**	**1.00*E*** + **10**	**1.00*E*** + **10**
	Std	**0.00*E*** + **00**	**0.00*E*** + **00**	**0.00*E*** + **00**	**0.00*E*** + **00**	**0.00*E*** + **00**

***F*4**	Mean	6.87*E* − 01	1.07*E* + 03	6.14*E* + 00	2.10*E* + 03	**3.97*E*** − **01**
	Std	5.74*E* − 01	3.42*E* + 02	5.00*E* + 00	8.76*E* + 02	**4.98*E*** − **01**

***F*5**	Mean	**0.00*E*** + **00**	**0.00*E*** + **00**	1.14*E* − 14	**0.00*E*** + **00**	**0.00*E*** + **00**
	Std	**0.00*E*** + **00**	**0.00*E*** + **00**	3.50*E* − 14	**0.00*E*** + **00**	**0.00*E*** + **00**

***F*6**	Mean	6.70*E* + 00	7.04*E* + 00	2.08*E* − 03	1.12*E* + 00	**1.36*E*** − **13**
	Std	1.17*E* + 01	1.05*E* + 01	6.29*E* − 03	2.63*E* + 00	**4.37*E*** − **13**

***F*7**	Mean	3.62*E* + 01	1.03*E* + 02	6.03*E* + 01	1.06*E* + 02	**3.53*E*** + **01**
	Std	1.43*E* + 01	2.15*E* + 01	2.85*E* + 01	2.27*E* + 01	**7.09*E*** + **00**

***F*8**	Mean	**2.08*E*** + **01**	2.09*E* + 01	2.09*E* + 01	2.09*E* + 01	2.09*E* + 01
	Std	9.27*E* − 02	**4.26*E* − 02**	4.27*E* − 02	5.48*E* − 02	5.64*E* − 02

***F*9**	Mean	2.42*E* + 01	2.70*E* + 01	2.61*E* + 01	2.89*E* + 01	**2.16*E*** + **01**
	Std	3.73*E* + 00	2.51*E* + 00	4.07*E* + 00	**1.45*E*** + **00**	2.68*E* + 00

***F*10**	Mean	1.03*E* − 01	1.24*E* − 02	**3.82*E*** − **03**	1.39*E* − 02	1.63*E* − 02
	Std	5.94*E* − 02	8.24*E* − 03	**4.91*E*** − **03**	1.14*E* − 02	1.49*E* − 02

***F*11**	Mean	1.95*E* + 01	8.06*E* + 00	**3.23*E*** + **00**	4.53*E* + 00	1.21*E* + 01
	Std	7.68*E* + 00	4.55*E* + 00	**1.51*E*** + **00**	3.62*E* + 00	3.20*E* + 00

***F*12**	Mean	6.16*E* + 01	1.31*E* + 02	1.18*E* + 02	1.62*E* + 02	**5.38*E*** + **01**
	Std	1.57*E* + 01	2.51*E* + 01	2.90*E* + 01	3.10*E* + 01	**1.12*E*** + **01**

***F*13**	Mean	1.13*E* + 02	1.58*E* + 02	1.44*E* + 02	1.80*E* + 02	**1.00*E*** + **02**
	Std	2.39*E* + 01	3.03*E* + 01	2.51*E* + 01	**2.30*E*** + **01**	2.62*E* + 01

***F*14**	Mean	1.70*E* + 03	1.28*E* + 03	8.96*E* + 02	7.41*E* + 02	1.02*E* + 03
	Std	4.70*E* + 02	3.23*E* + 02	3.04*E* + 02	**1.74*E*** + **02**	2.68*E* + 02

***F*15**	Mean	3.89*E* + 03	4.12*E* + 03	3.96*E* + 03	4.17*E* + 03	**3.46*E*** + **03**
	Std	5.76*E* + 02	3.75*E* + 02	5.93*E* + 02	**2.70*E*** + **02**	7.47*E* + 02

***F*16**	Mean	**6.84*E*** − **01**	9.30*E* − 01	1.52*E* + 00	1.42*E* + 00	1.38*E* + 00
	Std	4.53*E* − 01	**1.31*E*** − **01**	2.74*E* − 01	2.40*E* − 01	3.41*E* − 01

***F*17**	Mean	6.51*E* + 01	9.12*E* + 01	5.85*E* + 01	6.36*E* + 01	**5.29*E*** + **01**
	Std	1.20*E* + 01	1.35*E* + 01	**7.11*E*** + **00**	9.61*E* + 00	7.81*E* + 00

***F*18**	Mean	8.70*E* + 01	2.22*E* + 02	1.69*E* + 02	1.95*E* + 02	**8.25*E*** + **01**
	Std	**1.62*E*** + **01**	2.78*E* + 01	2.63*E* + 01	2.94*E* + 01	1.89*E* + 01

***F*19**	Mean	3.45*E* + 00	8.99*E* + 00	5.71*E* + 00	6.94*E* + 00	**2.29*E*** + **00**
	Std	1.16*E* + 00	1.44*E* + 00	2.08*E* + 00	1.75*E* + 00	**4.77*E*** − **01**

***F*20**	Mean	1.12*E* + 01	1.21*E* + 01	1.20*E* + 01	1.24*E* + 01	**1.11*E*** + **01**
	Std	5.63*E* − 01	**3.46*E*** − **01**	4.08*E* − 01	3.88*E* − 01	4.84*E* − 01

***F*21**	Mean	3.01*E* + 02	2.61*E* + 02	**2.05*E*** + **02**	2.44*E* + 02	2.47*E* + 02
	Std	9.54*E* + 01	4.66*E* + 01	**3.94*E*** + **01**	4.78*E* + 01	5.16*E* + 01

***F*22**	Mean	1.63*E* + 03	1.95*E* + 03	1.16*E* + 03	1.17*E* + 03	**1.04*E*** + **03**
	Std	8.40*E* + 02	5.17*E* + 02	4.25*E* + 02	**3.07*E*** + **02**	3.16*E* + 02

***F*23**	Mean	4.04*E* + 03	4.81*E* + 03	5.07*E* + 03	5.11*E* + 03	**3.68*E*** + **03**
	Std	4.98*E* + 02	4.42*E* + 02	5.54*E* + 02	**4.41*E*** + **02**	5.32*E* + 02

***F*24**	Mean	2.65*E* + 02	2.76*E* + 02	2.67*E* + 02	2.81*E* + 02	**2.59*E*** + **02**
	Std	8.67*E* + 00	**6.06*E*** + **00**	1.39*E* + 01	1.07*E* + 01	7.22*E* + 00

***F*25**	Mean	2.75*E* + 02	2.91*E* + 02	2.83*E* + 02	3.00*E* + 02	**2.73*E*** + **02**
	Std	1.07*E* + 01	6.77*E* + 00	1.08*E* + 01	**4.68*E*** + **00**	6.45*E* + 00

***F*26**	Mean	2.16*E* + 02	**2.00*E*** + **02**	**2.00*E*** + **02**	**2.00*E*** + **02**	**2.00*E*** + **02**
	Std	4.84*E* + 01	2.41*E* − 02	3.14*E* − 03	1.43*E* − 02	**1.57*E*** − **04**

***F*27**	Mean	8.79*E* + 02	1.05*E* + 03	9.47*E* + 02	1.03*E* + 03	**8.57*E*** + **02**
	Std	1.21*E* + 02	**5.27*E*** + **01**	2.04*E* + 02	2.20*E* + 02	1.42*E* + 02

***F*28**	Mean	3.00*E* + 02	**3.00*E*** + **02**	**3.00*E*** + **02**	**3.00*E*** + **02**	**3.00*E*** + **02**
	Std	1.04*E* − 13	**0.00*E*** + **00**	**0.00*E*** + **00**	**0.00*E*** + **00**	**0.00*E*** + **00**

**Average ranking**		2.18	3.43	2.50	3.36	1.46
+/−/≈		22/2/4	19/3/6	19/4/5	18/4/6	

In the table, bold letters indicate the best results, “Mean” and “Std” represent the mean and standard error values, respectively.

**Table 4:** The test result of CEC2013 of MSRCS, VCA, CS, ACS, ICS, and GFCS on CEC2013 (*D* = 50).

Function	Mean/std	MSRCS	VCS	CS	ACS	ICS	GFCS
***F*1**	Mean	**0.00*E*** + **00**	**0.00*E*** + **00**	**0.00*E*** + **00**	**0.00*E*** + **00**	**0.00*E*** + **00**	**0.00*E*** + **00**
	Std	**0.00*E*** + **00**	**0.00*E*** + **00**	**0.00*E*** + **00**	**0.00*E*** + **00**	**0.00*E*** + **00**	**0.00*E*** + **00**

***F*2**	Mean	1.99*E* + 05	6.35*E* + 06	3.58*E* + 06	4.01*E* + 06	8.87*E* + 05	**1.37*E*** + **05**
	Std	8.87*E* + 04	1.74*E* + 06	9.78*E* + 05	8.16*E* + 05	2.02*E* + 05	**2.48*E*** + **03**

***F*3**	Mean	**1.00*E*** + **10**	**1.00*E*** + **10**	**1.00*E*** + **10**	**1.00*E*** + **10**	**1.00*E*** + **10**	**1.00*E*** + **10**
	Std	**0.00*E*** + **00**	**0.00*E*** + **00**	**0.00*E*** + **00**	**0.00*E*** + **00**	**0.00*E*** + **00**	**0.00*E*** + **00**

***F*4**	Mean	**6.40*E*** + **01**	5.29*E* + 03	7.79*E* + 03	5.34*E* + 03	6.01*E* + 02	1.07*E* + 02
	Std	**4.70*E*** + **01**	1.16*E* + 03	1.50*E* + 03	2.22*E* + 03	2.04*E* + 02	4.91*E* + 01

***F*5**	Mean	1.14*E* − 13	1.14*E* − 13	5.68*E* − 14	7.96*E* − 14	**1.02*E*** − **13**	1.06*E* − 13
	Std	**0.00*E*** + **00**	**0.00*E*** + **00**	5.99*E* − 14	5.49*E* − 14	3.60*E* − 14	2.94*E* − 14

***F*6**	Mean	4.46*E* + 01	4.40*E* + 01	4.44*E* + 01	**4.09*E*** + **01**	4.40*E* + 01	4.38*E* + 01
	Std	2.39*E* + 00	**1.80*E*** + **00**	1.70*E* + 01	1.02*E* + 01	**1.80*E*** + **00**	1.47*E* + 00

***F*7**	Mean	7.62*E* + 01	1.05*E* + 02	1.23*E* + 02	1.14*E* + 02	9.54*E* + 01	**7.53*E*** + **01**
	Std	1.75*E* + 01	1.59*E* + 01	1.63*E* + 01	1.72*E* + 01	2.74*E* + 01	**1.26*E*** + **01**

***F*8**	Mean	**2.10*E*** + **01**	2.11*E* + 01	2.11*E* + 01	2.11*E* + 01	2.11*E* + 01	2.11*E* + 01
	Std	6.27*E* − 02	2.95*E* − 02	**2.22*E*** − **02**	2.84*E* − 02	4.96*E* − 02	2.36*E* − 02

***F*9**	Mean	5.12*E* + 01	5.68*E* + 01	5.81*E* + 01	5.49*E* + 01	5.64*E* + 01	**4.94*E* + 01**
	Std	6.36*E* + 00	**1.47*E*** + **00**	2.10*E* + 00	3.22*E* + 00	3.49*E* + 00	3.98*E* + 00

***F*10**	Mean	1.10*E* − 01	4.56*E* − 2	5.75*E* − 02	5.30*E* − 02	**2.69*E*** − **02**	6.03*E* − 02
	Std	6.13*E* − 02	2.65*E* − 02	3.73*E* − 02	**1.98*E*** − **02**	3.24*E* − 02	3.12*E* − 02

***F*11**	Mean	6.83*E* + 01	4.29*E* + 01	3.22*E* + 01	5.43*E* + 01	**3.13*E*** + **01**	5.01*E* + 01
	Std	1.47*E* + 01	1.25*E* + 01	7.68*E* + 00	1.72*E* + 01	**7.54*E*** + **00**	9.25*E* + 00

***F*12**	Mean	1.80*E* + 02	2.03*E* + 02	3.99*E* + 02	3.70*E* + 02	3.55*E* + 02	**1.37*E*** + **02**
	Std	3.09*E* + 01	2.79*E* + 01	6.07*E* + 01	6.24*E* + 01	7.74*E* + 01	**2.30*E*** + **01**

***F*13**	Mean	2.65*E* + 02	2.94*E* + 02	4.73*E* + 02	4.08*E* + 02	4.18*E* + 02	**2.50*E*** + **02**
	Std	3.92*E* + 01	3.97*E* + 01	8.72*E* + 01	**3.91*E*** + **01**	4.27*E* + 01	4.04*E* + 01

***F*14**	Mean	3.49*E* + 03	**2.06*E*** + **03**	2.32*E* + 03	4.48*E* + 03	2.39*E* + 03	2.49*E* + 03
	Std	5.35*E* + 02	7.55*E* + 02	4.70*E* + 02	6.53*E*+02	5.51*E* + 02	**4.00*E*** + **02**

***F*15**	Mean	**7.91*E*** + **03**	9.48*E* + 03	8.51*E* + 03	8.48*E* + 03	9.19*E* + 03	8.19*E* + 03
	Std	7.64*E* + 02	5.39*E* + 02	4.79*E* + 02	**3.52*E*** + **02**	4.16*E* + 02	1.32*E* + 03

***F*16**	Mean	**9.56*E*** − **01**	2.24*E* + 00	2.36*E* + 00	1.70*E* + 00	2.51*E* + 00	2.25*E* + 00
	Std	4.04*E* − 01	2.04*E* − 01	2.14*E* − 01	**1.75*E*** − **01**	2.12*E* − 01	4.81*E* − 01

***F*17**	Mean	1.55*E* + 02	**1.23*E*** + **02**	1.96*E* + 02	3.02*E* + 02	1.50*E* + 02	1.27*E* + 02
	Std	3.73*E* + 01	**1.20*E*** + **01**	3.01*E* + 01	3.98*E* + 01	2.81*E* + 01	1.87*E* + 01

***F*18**	Mean	**1.85*E*** + **02**	3.20*E* + 02	5.04*E* + 02	6.05*E* + 02	4.21*E* + 02	2.99*E* + 02
	Std	**2.49*E*** + **01**	3.16*E* + 01	8.35*E* + 01	6.32*E* + 01	8.50*E* + 01	8.86*E* + 01

***F*19**	Mean	8.36*E* + 00	1.17*E* + 01	2.82*E* + 01	3.01*E* + 01	1.52*E* + 01	**5.67*E*** + **00**
	Std	**1.03*E*** + **00**	4.43*E* + 00	7.78*E* + 00	3.14*E* + 00	3.25*E* + 00	1.39*E* + 00

***F*20**	Mean	**2.06*E*** + **01**	2.18*E* + 01	2.24*E* + 01	2.19*E* + 01	2.20*E* + 01	2.09*E* + 01
	Std	8.43*E* − 01	5.08*E* − 01	7.66*E* − 01	**4.01*E*** − **01**	7.31*E* − 01	5.90*E* − 01

***F*21**	Mean	9.16*E* + 02	6.02*E* + 02	6.04*E* + 02	5.52*E* + 02	4.48*E* + 02	**3.46*E*** + **02**
	Std	**2.89*E*** + **02**	4.36*E* + 02	4.38*E* + 02	4.38*E* + 02	4.07*E* + 02	3.09*E* + 02

***F*22**	Mean	3.90*E* + 03	**1.98*E*** + **03**	3.76*E* + 03	5.27*E* + 03	3.11*E* + 03	2.65*E* + 03
	Std	9.09*E* + 02	**8.13*E*** + **02**	8.16*E* + 02	1.06*E* + 03	8.90*E* + 02	1.12*E* + 03

***F*23**	Mean	**7.91*E*** + **03**	1.06*E* + 04	1.04*E* + 04	1.00*E* + 04	1.10*E* + 04	9.12*E* + 03
	Std	1.06*E* + 03	6.87*E* + 02	**5.56*E*** + **02**	8.50*E* + 02	8.63*E* + 02	1.54*E* + 03

***F*24**	Mean	**3.08*E*** + **02**	3.52*E* + 02	3.68*E* + 02	3.49*E* + 02	3.39*E* + 02	3.21*E* + 02
	Std	1.76*E* + 01	**9.07*E*** + **00**	1.37*E* + 01	1.68*E* + 01	2.62*E* + 01	1.51*E* + 01

***F*25**	Mean	**3.36*E*** + **02**	3.76*E* + 02	3.98*E* + 02	3.82*E* + 02	3.80*E* + 02	3.65*E* + 02
	Std	1.22*E* + 01	3.41*E* + 00	**6.96*E*** + **00**	1.40*E* + 01	1.28*E* + 01	1.17*E* + 01

***F*26**	Mean	2.48*E* + 02	2.25*E* + 02	**2.00*E*** + **02**	2.01*E* + 02	**2.00*E*** + **02**	**2.00*E*** + **02**
	Std	1.02*E* + 02	7.71*E* + 01	1.09*E* − 01	1.76*E* − 01	2.49*E* − 02	**3.17*E*** − **03**

***F*27**	Mean	1.55*E* + 03	1.82*E* + 03	1.92*E* + 03	1.79*E* + 03	1.78*E* + 03	**1.54*E*** + **03**
	Std	1.59*E* + 02	**2.96*E*** + **01**	4.95*E* + 01	8.82*E* + 01	2.19*E* + 02	9.50*E* + 01
***F*28**	Mean	7.21*E* + 02	**4.00*E*** + **02**	7.30*E* + 02	1.04*E* + 03	**4.00*E*** + **02**	**4.00*E*** + **02**
	Std	1.02*E* + 03	**0.00*E*** + **00**	1.04*E* + 03	1.35*E* + 03	1.33*E* − 13	**0.00*E*** + **00**

**Average ranking**		2.79	3.29	4.25	3.93	3.11	1.96
+/−/≈		17/2/9	18/3/7	20/3/5	21/3/4	18/6/4	

In the table, bold letters indicate the best results, “Mean” and “Std” represent the mean and standard error values, respectively.

**Table 5:** The test result of CEC2013 of DE, FA, NABC, ABCX, CUDE, MSBSO, and GFCS on CEC2013 (*D* = 30).

Function	Mean/std	DE	FA	NABC	ABCX	CUDE	MSBSO	GFCS
***F*1**	Mean	1.06*E* − 13	6.82*E* − 13	4.09*E* − 13	2.37*E* − 13	1.82*E* − 13	**0.00*E*** + **00**	**0.00*E*** + **00**
	Std	1.17*E* − 13	1.61*E* − 13	9.59*E* − 14	7.15*E* − 14	1.44*E* − 13	**0.00*E*** + **00**	**0.00*E*** + **00**

***F*2**	Mean	4.10*E* + 07	4.55*E* + 04	2.00*E* + 06	1.76*E* + 07	8.16*E* + 03	5.74*E* + 04	**1.96*E*** + **03**
	Std	9.32*E* + 06	3.21*E* + 04	5.24*E* + 05	3.01*E* + 06	4.97*E* + 03	1.67*E* + 04	**2.48*E*** + **03**

***F*3**	Mean	3.38*E* + 07	1.82*E* + 08	2.74*E* + 07	8.99*E* + 08	**3.69*E*** + **05**	1.04*E* + 06	1.00*E* + 10
	Std	3.93*E* + 07	3.07*E* + 08	1.76*E* + 07	9.89*E* + 07	6.62*E* + 05	1.18*E* + 06	**0.00*E*** + **00**

***F*4**	Mean	6.85*E* + 03	3.34*E* + 02	5.19*E* + 04	6.12*E* + 04	**6.43*E*** − **10**	9.19*E* − 03	3.97*E* − 01
	Std	1.14*E* + 03	3.73*E* + 02	5.34*E* + 03	6.03*E* + 03	**1.64*E*** − **09**	1.21*E* − 02	4.98*E* − 01

***F*5**	Mean	1.14*E* − 13	2.27*E* − 12	6.03*E* − 13	2.72*E* − 13	2.73*E* − 13	**0.00*E*** + **00**	**0.00*E*** + **00**
	Std	**0.00*E*** + **00**	1.73*E* − 12	9.36*E* − 14	6.72*E* − 14	5.87*E* − 14	**0.00*E*** + **00**	**0.00*E*** + **00**

***F*6**	Mean	1.43*E* + 01	1.38*E* + 01	1.46*E* + 01	1.49*E* + 01	3.32*E* + 00	1.09*E* + 01	**1.36*E*** − **13**
	Std	4.53*E* + 00	1.10*E* − 11	7.04*E* − 01	4.69*E* + 00	6.15*E* + 00	1.08*E* + 01	**4.37*E*** − **13**

***F*7**	Mean	3.89*E* + 01	7.13*E* + 01	6.01*E* + 01	8.43*E* + 01	3.97*E* + 01	**1.07*E*** + **01**	3.53*E* + 01
	Std	1.16*E* + 01	1.30*E* + 01	7.26*E* + 00	9.02*E* + 00	1.09*E* + 01	**7.00*E*** + **00**	7.09*E* + 00

***F*8**	Mean	2.09*E* + 01	**2.08*E*** + **01**	2.09*E* + 01	2.09*E* + 01	2.09*E* + 01	2.09*E* + 01	2.09*E* + 01
	Std	**2.87*E*** − **02**	5.41*E* − 02	3.16*E* − 02	5.28*E* − 02	4.48*E* − 02	5.44*E* − 02	5.64*E* − 02

***F*9**	Mean	3.16*E* + 01	**2.07*E*** + **01**	2.59*E* + 01	2.71*E* + 01	2.23*E* + 01	2.45*E* + 01	2.16*E* + 01
	Std	**1.34*E*** + **00**	2.49*E* + 00	3.79*E* + 00	1.99*E* + 00	3.99*E* + 00	5.94*E*+00	2.68*E* + 00

***F*10**	Mean	5.07*E* − 01	1.85*E* − 01	7.00*E* − 02	1.41*E* + 00	1.21*E* − 01	4.66*E* − 02	**1.63*E*** − **02**
	Std	6.21*E* − 01	8.20*E* − 02	7.74*E* − 02	2.20*E* − 01	5.24*E* − 02	2.68*E* − 02	**1.49*E*** − **02**

***F*11**	Mean	3.32*E* − 01	7.32*E* + 01	**5.68*E*** − **14**	6.19*E* − 14	9.05*E* + 00	2.31*E* + 01	1.21*E* + 01
	Std	6.14*E* − 01	2.26*E* + 01	**0.00*E*** + **00**	1.65*E* − 14	7.66*E* + 00	4.49*E* + 00	3.20*E* + 00

***F*12**	Mean	1.50*E* + 02	8.58*E* + 01	1.42*E* + 02	2.29*E* + 02	6.83*E* + 01	**3.84*E*** + **01**	5.38*E* + 01
	Std	1.13*E* + 01	2.24*E* + 01	2.64*E* + 01	1.59*E* + 01	1.62*E* + 01	**9.20*E*** + **00**	1.12*E* + 01

***F*13**	Mean	1.58*E* + 02	1.54*E* + 02	2.01*E* + 02	2.00*E* + 02	1.12*E* + 02	**9.31*E*** + **01**	1.00*E* + 02
	Std	1.51*E* + 01	4.33*E* + 01	3.00*E* + 01	**1.04*E*** + **01**	2.43*E* + 01	1.69*E* + 01	2.62*E* + 01

***F*14**	Mean	1.62*E* + 02	2.20*E* + 03	**3.27*E*** − **01**	4.03*E* − 02	1.69*E* + 01	4.37*E* + 02	1.02*E* + 03
	Std	9.94*E* + 01	6.46*E* + 02	**3.32*E*** − **01**	4.23*E* − 02	3.78*E* + 01	2.15*E* + 02	2.68*E* + 02

***F*15**	Mean	6.89*E* + 03	3.62*E* + 03	**2.99*E*** + **03**	4.21*E* + 03	3.84*E* + 03	3.67*E* + 03	3.46*E* + 03
	Std	3.50*E* + 02	6.47*E* + 02	8.31*E* + 02	**2.80*E*** + **02**	5.55*E* + 02	6.38*E* + 02	7.47*E* + 02

***F*16**	Mean	2.31*E* + 00	1.89*E* + 00	**6.69*E*** − **01**	1.62*E* + 00	9.01*E* − 01	2.09*E* + 00	1.38*E* + 00
	Std	3.01*E* − 01	**2.28*E*** − **01**	2.35*E* − 01	2.77*E* − 01	5.00*E* − 01	3.82*E* − 01	3.41*E* − 01

***F*17**	Mean	3.07*E* + 01	8.09*E* + 01	3.04*E* + 01	**3.03*E*** + **01**	3.18*E* + 01	5.69*E* + 01	5.29*E* + 01
	Std	3.11*E* − 01	1.13*E* + 01	**5.09*E*** − **03**	5.88*E* − 03	2.66*E* + 00	8.30*E* + 00	7.81*E* + 00

***F*18**	Mean	2.05*E* + 02	8.38*E* + 01	1.97*E* + 02	1.52*E* + 02	8.59*E* + 01	**6.29*E*** + **01**	8.25*E* + 01
	Std	**8.95*E*** + **00**	5.06*E* + 01	3.36*E* + 01	1.56*E* + 01	2.95*E* + 01	9.01*E* + 00	1.89*E* + 01

***F*19**	Mean	4.27*E* + 00	3.40*E* + 00	**5.14*E*** − **02**	3.28*E* − 01	3.33*E* + 00	3.11*E* + 00	2.29*E* + 00
	Std	3.72*E* − 01	5.52*E* − 01	**4.54*E*** − **02**	2.23*E* − 01	2.19*E* + 00	9.72*E* − 01	4.77*E* − 01

***F*20**	Mean	1.24*E* + 01	1.50*E* + 01	1.47*E*+01	1.37*E* + 01	1.01*E* + 01	**9.71*E*** + **00**	1.11*E* + 01
	Std	2.59*E* − 01	**0.00*E*** + **00**	7.24*E* − 01	3.98*E* − 01	8.52*E* − 01	7.59*E* − 01	4.84*E* − 01

***F*21**	Mean	2.96*E* + 02	3.57*E* + 02	3.76*E*+02	**2.03*E*** + **02**	3.47*E* + 02	3.04*E* + 02	2.47*E* + 02
	Std	**2.79*E*** + **01**	7.86*E* + 01	9.17*E* + 01	4.42*E* + 01	8.82*E* + 01	5.81*E* + 01	5.16*E* + 01

***F*22**	Mean	1.50*E* + 02	2.23*E* + 03	8.46*E* + 01	**5.67*E*** + **01**	1.23*E* + 02	4.39*E* + 02	1.04*E* + 03
	Std	3.41*E* + 01	8.12*E* + 02	4.13*E* + 01	4.83*E* + 01	**1.13*E*** + **01**	1.45*E* + 02	3.16*E* + 02

***F*23**	Mean	7.09*E* + 03	3.89*E* + 03	4.39*E* + 03	**5.01*E*** + **01**	3.87*E* + 03	4.16*E* + 03	3.68*E* + 03
	Std	3.84*E* + 02	8.23*E* + 02	7.12*E* + 02	**3.58*E*** + **02**	6.47*E* + 02	6.81*E* + 02	5.32*E* + 02

***F*24**	Mean	2.76*E* + 02	2.55*E* + 02	2.31*E* + 02	2.63*E* + 02	2.31*E* + 02	**2.19*E*** + **02**	2.59*E* + 02
	Std	4.51*E* + 00	1.00*E* + 01	**2.66*E*** + **00**	7.45*E* + 00	3.95*E* + 00	7.63*E* + 00	7.22*E* + 00

***F*25**	Mean	2.83*E* + 02	2.70*E* + 02	**2.59*E*** + **02**	2.92*E* + 02	2.72*E* + 02	2.62*E* + 02	2.75*E* + 02
	Std	4.58*E* + 00	7.87*E* + 00	4.13*E* + 01	**4.23*E*** + **00**	2.04*E* + 01	8.13*E* + 00	6.45*E* + 00

***F*26**	Mean	2.16*E* + 02	**2.00*E*** + **02**	**2.00*E*** + **02**	2.01*E* + 02	2.12*E* + 02	2.11*E* + 02	**2.00*E*** + **02**
	Std	4.49*E* + 01	4.20*E* − 03	1.66*E* − 02	1.54*E* − 01	3.79*E* + 01	3.56*E* + 01	**1.57*E*** − **04**

***F*27**	Mean	1.08*E* + 03	7.65*E* + 02	5.49*E* + 02	**4.02*E*** + **02**	6.41*E* + 02	6.07*E* + 02	8.57*E* + 02
	Std	3.50*E* + 01	5.78*E* + 01	1.93*E* + 02	1.44*E* + 00	**1.18*E*** + **02**	1.75*E* + 02	1.42*E* + 02

***F*28**	Mean	3.00*E* + 02	5.21*E* + 02	3.00*E* + 02	**2.83*E*** + **02**	4.12*E* + 02	3.00*E* + 02	3.00*E* + 02
	Std	1.52*E* − 13	4.93*E* + 02	2.48*E* − 13	5.69*E* + 01	3.55*E* + 02	2.14*E* − 13	**0.00*E*** + **00**
**Average ranking**		5.07	4.79	3.71	4.25	3.43	3.03	3.00
+/−/≈		21/2/5	20/2/6	14/3/11	18/1/9	16/1/11	12/4/12	

In the table, bold letters indicate the best results, “Mean” and “Std” represent the mean and standard error values, respectively.

**Table 6:** The test result of CEC2013 of GA, ACO, ABC, BSO, and GFCS on CEC2013 (*D* = 30).

Function	Mean/std	GA	PSO	ACO	ABC	BSO	GFCS
***F*1**	Mean Std	1.55*E* − 07 1.14*E* − 07	4.00*E* − 05 6.05*E* − 05	1.40*E* − 02 1.77*E* − 03	5.23*E* − 13 1.53*E* − 13	1.32*E* − 02 1.23*E* + 00	**0.00*E*** + **00** **0.00*E*** + **00**
***F*2**	Mean Std	4.08*E* + 06 3.18*E* + 06	1.64*E* + 06 9.48*E* + 05	1.61*E* + 06 7.01*E* + 05	1.12*E* + 08 1.43*E* + 07	8.92*E* + 05 3.66*E* + 05	**1.96*E*** + **03** **2.48*E*** + **03**
***F*3**	Mean Std	8.21*E* + 09 4.11*E* + 09	4.04*E* + 08 5.77*E* + 08	3.68*E* + 08 4.76*E* + 08	**9.40*E*** + **01** **6.61*E*** + **01**	1.29*E* + 08 2.38*E* + 08	1.00*E* + 10 0.00*E* + 00
***F*4**	Mean Std	8.10*E* + 04 2.52*E* + 04	4.38*E* + 00 2.61*E* + 00	1.07*E* + 03 3.42*E* + 02	6.52*E* + 04 1.31*E* + 04	1.10*E* + 03 9.76*E* + 02	**3.97*E*** − **01** **4.98*E*** − **01**
***F*5**	Mean Std	3.20*E* + 02 2.25*E* + 02	2.00*E* − 05 6.12*E* − 05	5.71*E* + 04 1.43*E* + 04	1.09*E* − 03 2.96*E* − 04	5.33*E* − 02 1.38*E* − 02	**0.00*E*** + **00** **0.00*E*** + **00**
***F*6**	Mean Std	8.36*E* + 01 2.04*E* + 01	5.72*E* + 01 2.73*E* + 01	5.05*E* + 00 2.66*E* + 00	1.39*E* + 01 6.96*E* − 02	3.12*E* + 01 2.63*E* + 01	**1.36*E*** − **13** **4.37*E*** − **13**
***F*7**	Mean Std	3.28*E* + 04 5.29*E* + 04	1.01*E* + 02 1.52*E* + 01	1.15*E* + 02 1.80*E* + 01	7.09*E* + 01 **3.05*E*** + **00**	1.06*E* + 02 5.27*E* + 01	**3.53*E*** + **01** 7.09*E* + 00
***F*8**	Mean Std	2.10*E* + 01 6.37*E* − 02	**2.09*E*** + **01** 5.51*E* − 02	**2.09*E*** + **01** 6.08*E* − 02	**2.09*E*** + **01** **3.08*E*** − **02**	**2.09*E*** + **01** 1.48*E* − 01	**2.09*E*** + **01** 5.64*E* − 02
***F*9**	Mean Std	3.63*E* + 01 4.05*E* + 00	2.76*E* + 01 2.31*E* + 00	2.31*E* + 01 1.07*E* + 00	3.85*E* + 01 **9.03*E*** − **01**	3.89*E* + 01 2.45*E* + 00	**2.16*E*** + **01** 2.68*E* + 00
***F*10**	Mean Std	5.93*E* + 00 5.65*E* + 00	2.63*E* + 00 2.61*E* + 00	2.42*E* − 01 3.00*E* − 02	**1.50*E*** − **03** **3.11*E*** − **03**	1.03*E* + 00 1.14*E* − 01	1.63*E* − 02 1.49*E* − 02
***F*11**	Mean Std	3.90*E* + 02 1.07*E* + 02	1.18*E* + 02 3.02*E* + 01	2.88*E* + 02 8.09*E* + 01	1.87*E* + 02 1.13*E* + 01	5.53*E* + 02 1.62*E* + 02	**1.21*E*** + **01** **3.20*E*** + **00**
***F*12**	Mean Std	3.07*E* + 02 4.81*E* + 01	1.71*E* + 02 5.48*E* + 01	2.51*E* + 02 6.52*E* + 01	1.98*E* + 02 1.47*E* + 01	6.62*E* + 02 9.10*E* + 01	**5.38*E*** + **01** **1.12*E*** + **01**
***F*13**	Mean Std	4.02*E* + 02 3.67*E* + 01	2.62*E* + 02 6.05*E* + 01	3.96*E* + 02 8.17*E* + 01	2.01*E* + 02 **1.32*E*** + **01**	6.80*E* + 02 8.30*E* + 01	**1.00*E*** + **02** 2.62*E* + 01
***F*14**	Mean Std	3.98*E* + 03 3.46*E* + 02	3.32*E* + 03 7.04*E* + 02	2.76*E* + 03 5.81*E* + 02	6.50*E* + 03 2.86*E* + 02	4.41*E* + 03 6.74*E* + 02	**1.02*E*** + **03** **2.68*E*** + **02**
***F*15**	Mean Std	4.49*E* + 03 6.58*E* + 02	4.39*E* + 03 8.03*E* + 02	**2.94*E*** + **03** 3.62*E* + 02	7.25*E* + 03 **3.03*E*** + **02**	4.17*E* + 03 7.30*E* + 02	3.46*E* + 03 7.47*E* + 02
***F*16**	Mean Std	3.11*E* + 00 9.06*E* − 01	1.16*E* + 00 5.13*E* − 01	**1.84*E*** − **01** **4.21*E*** − **02**	2.18*E* + 00 2.95*E* − 01	3.42*E* − 01 1.40*E* − 01	1.38*E* + 00 3.41*E* − 01
***F*17**	Mean Std	4.70*E* + 02 1.11*E* + 02	1.29*E* + 02 3.94*E* + 01	4.98*E* + 02 9.58*E* + 01	2.32*E* + 02 1.46*E* + 01	5.36*E* + 02 9.61*E* + 00	**5.29*E*** + **01** **7.81*E*** + **00**
***F*18**	Mean Std	4.14*E* + 02 1.20*E* + 02	1.37*E* + 02 4.63*E* + 01	4.13*E* + 02 4.94*E* + 01	2.43*E* + 02 **1.60*E*** + **01**	4.95*E* + 02 7.94*E* + 01	**8.25*E*** + **01** 1.89*E* + 01
***F*19**	Mean Std	3.65*E* + 02 6.07*E* + 01	4.97*E* + 00 2.09*E* + 00	1.91*E* + 01 6.13*E* + 00	1.88*E* + 01 6.31*E* − 01	1.14*E* + 01 2.75*E* + 00	**2.29*E*** + **00** **4.77*E*** − **01**
***F*20**	Mean Std	1.46*E* + 01 2.22*E* − 01	1.44*E* + 01 5.77*E* − 01	1.45*E* + 01 5.33*E* − 03	1.28*E* + 01 **1.46*E*** − **01**	1.44*E* + 01 3.88*E* − 02	**1.11*E*** + **01** 4.84*E* − 01
***F*21**	Mean Std	2.80*E* + 02 4.47*E* + 01	3.42*E* + 02 1.23*E* + 02	3.02*E* + 02 **7.98*E*** − **02**	2.80*E* + 02 4.22*E* + 01	3.74*E* + 02 1.08*E* + 02	**2.47*E*** + **02** 5.16*E* + 01
***F*22**	Mean Std	4.84*E* + 03 1.13*E* + 03	4.10*E* + 03 7.08*E* + 02	3.84*E* + 03 4.11*E* + 02	7.08*E* + 03 **2.48*E*** + **02**	5.17*E* + 03 1.07*E* + 03	**1.04*E*** + **03** 3.16*E* + 02
***F*23**	Mean Std	5.62*E* + 03 9.48*E* + 02	5.25*E* + 03 1.01*E* + 03	3.74*E* + 03 6.66*E* + 02	7.36*E* + 03 **3.11*E*** + **02**	5.11*E* + 03 7.41*E* + 02	**3.68*E*** + **03** 5.32*E* + 02
***F*24**	Mean Std	3.47*E* + 02 1.29*E* + 01	2.75*E* + 02 1.22*E* + 01	2.82*E* + 02 **3.07*E*** + **00**	2.93*E* + 02 3.24*E* + 00	3.61*E* + 02 2.17*E* + 02	**2.59*E*** + **02** 7.22*E* + 00
***F*25**	Mean Std	3.78*E* + 02 1.43*E* + 01	3.00*E* + 02 1.30*E* + 01	3.05*E* + 02 5.82*E* + 00	3.02*E* + 02 **1.92*E*** + **00**	3.54*E* + 02 2.21*E* + 01	**2.73*E*** + **02** 6.45*E* + 00
***F*26**	Mean Std	3.92*E* + 02 7.58*E* + 00	3.29*E* + 02 6.79*E* + 01	**2.00*E*** + **02** 7.68*E* − 03	2.21*E* + 02 3.45*E* + 00	2.20*E* + 02 5.13*E* + 01	**2.00*E*** + **02** **1.57*E*** − **04**
***F*27**	Mean Std	1.32*E* + 03 6.71*E* + 01	1.03*E* + 03 8.74*E* + 01	8.93*E* + 02 6.50*E* + 01	1.26*E* + 03 **2.06*E*** + **01**	1.32*E* + 03 8.20*E* + 01	**8.57*E*** + **02** 1.42*E* + 02
***F*28**	Mean Std	3.76*E* + 03 5.71*E* + 02	4.04*E* + 02 3.94*E* + 02	9.15*E* + 02 6.98*E* + 02	**3.00*E*** + **02** 7.90*E* − 06	4.70*E* + 03 6.40*E* + 02	**3.00*E*** + **02** **0.00*E*** + **00**
**Average ranking**		5.14	3.14	3.25	3.46	4.14	1.39
+/−/≈		27/0/1	25/1/2	23/1/4	25/1/2	25/1/2	

In the tables, bold letters indicate the best results, “Mean” and “Std” represent the mean and standard error values, respectively.

**Table 7:** The runtime of CS and GFCS on CEC2013 (unit: seconds).

Dimension/algorithm	CS	GFCS
*D* = 30	5.483*E* + 03	5.443*E* + 03
*D* = 50	8.645*E* + 03	8.652*E* + 03

## Data Availability

The datasets generated and/or analysed in this study can be obtained from the corresponding authors upon reasonable request.
